# Clinical outcomes of dental implants placed in the same region where previous implants failed due to peri-implantitis: a retrospective study

**DOI:** 10.1186/s40729-021-00392-1

**Published:** 2021-11-09

**Authors:** Eduardo Anitua, Adriana Montalvillo, Asier Eguia, Mohammad Hamdan Alkhraisat

**Affiliations:** 1 University Institute for Regenerative Medicine and Oral Implantology - UIRMI (UPV/EHU-Fundación Eduardo Anitua), Jose Maria Cagigal 19, 01007 Vitoria, Spain; 2 BTI Biotechnology Institute, Vitoria, Spain

**Keywords:** Peri-implantitis, Dental implant, Survival, Implant supported dental prosthesis, Infection

## Abstract

**Purpose:**

There is paucity in the studies that assess dental implants replacing failed dental implants due to peri-implantitis. This study aims to evaluate the clinical outcomes of these implants in terms of implant survival and marginal bone loss.

**Methods:**

Patients in this retrospective study were selected if having one or more implants removed due to peri-implantitis and the placement and loading of dental implants in the same region from April 2010 to December 2019. Information was collected about the patient's demographic data, implant dimensions, surgical and prosthetic variables. Changes in peri-implant bone level, cumulative implant survival rate and technical complications were assessed.

**Results:**

Three hundred and eighty one dental implants in 146 patients that were placed in the same position or one-tooth position mesially/distally to the site of explantation were included. The patients' mean age was 63 ± 10 years. Ninety seven patients were females and 49 were males. After a mean follow-up of 34 ± 17 months, two implants failed. The cumulative survival rate was 99%. The marginal bone loss was −0.1 ± 0.6. Immediate or delay replacement of the failed implant did not affect implant survival or marginal bone stability. All the prostheses were screw-retained and presented the following complications: ceramic chipping (3 events), resin tooth fracture (1 event) and prosthetic screw loosening (1 event).

**Conclusions:**

Dental implants replacing failed implants due to peri-implantitis would be an option in the management of peri-implantitis. They showed high survival rate and marginal bone stability.

## Background

Modern dental implant therapy has become a predictable and successful restorative option to restore missing teeth. Recent studies estimate a 10-year implant survival of 96.4% of implants, when using contemporary systems [1]. Peri-implantitis is the most frequent complication, occurring in up to 20% of patients and 30% of implants [2]. The actual prevalence of peri-implantitis can be difficult to assess, due to the heterogeneous diagnostic and case definition criteria [3]. At the 2017 World Workshop on Periodontology, Working Group 4 has presented case definition of peri-implant health (no bleeding on probing and no bone loss beyond initial bone remodeling) and peri-implant mucositis (bleeding on probing and no bone loss beyond initial bone remodeling) [4]. For epidemiological studies, the case definition of peri-implantitis has been bleeding and/or suppuration on gentle probing, increased probing depth compared to previous examinations and bone loss. The adoption of these case definitions will help to overcome the heterogeneity in diagnostic and case definition criteria between studies.

The relevance and prevalence of peri-implantitis have increased as the number of implants placed and the longevity of the population have risen too [1–4]. This situation has reinforced the need for research on the causes of implant failure, implant removal techniques and the outcomes of reinsertion of dental implants in previous failed sites.

There is no evidence-based unanimously accepted treatment protocol for advanced peri-implantitis [5]. The treatment should be tailored to the severity of the lesion, which ranges from mechanical debridement to implant removal [5].

Failed implant sites sometimes present a challenge for clinicians, because the alveolar bone is usually further reduced, resulting in greater difficulties to place a second set of implants [6]. This can be more critical, when the cause of implant failure is peri-implantitis resulting in a great bone defect [7]. Second implantation after early and/or late failure has demonstrated with some limited strength of evidence a moderate survival rate, with a weighted rate close to 89% of implants for implants placed in sites with a history of one failure and 67.1% in sites with a history of two implant failures [8]. Nevertheless the existing studies present certain heterogeneity of methodology and results.

Most of the previous articles in literature in this topic were focused on the prognosis of dental implants analyzing data from early and/or late failure, independently of the implant retrieval cause [8]. In contrast, this work aimed to answer more specifically the following clinical question: What is the survival and complication of dental implants placed in the same region, where previous implants were failed due to peri-implantitis? This information could help clinicians to face the choice of treatment for advanced peri-implantitis. In contrast to previous research, this study aims to analyze the outcomes of dental implants placed in the same region of a failed dental implant due to peri-implantitis.

## Methods

This study was reported according to the STROBE (Strengthening the Reporting of Observational studies in Epidemiology) guidelines [9]. All procedures performed involving human participants were in accordance with the ethical standards of the institutional and/or national research committee and with the 1964 Helsinki declaration and its later amendments. The study was approved by the Ethical Committee of the Araba University Hospital (Expte. 2020-061). The study was performed in a private dental centre (Vitoria, Spain) to include patients who were surgically treated to remove a dental implant between April 2010 and December 2019.

The study is an observational retrospective study in which patients fulfilled the following criteria:

### Inclusion criteria

1. Had an age > 18 years
2. Had one or more implants removed due to peri-implantitis.
3. Placement and loading of dental implant in the same region as the explanted implant.

### Exclusion criteria

1. Had implant failed due to other causes different than peri-implantitis
2. Had the dental implants unloaded.
3. Smokers of > 10 cigarettes per day.
4. Had uncontrolled diabetes mellitus.
5. Had a follow-up time < 12 months.

### Surgical procedures

The cause of implant failure was judged to be peri-implantitis when the bone level was ≥ 3 mm below the most coronal intra-osseous part of the implant.

Implant removal was performed as explained elsewhere using an implant removal kit (Biotechnology Institute S.L, Vitoria, Spain) [4]. Briefly, the kit provided a ratchet that was engaged into the implant connection and then a counter-torque was exerted by a wrench. The wrench provided a maximum torque of 200 Ncm. If the torque needed to extract the implant was higher than 200 Ncm, trephine bur was used to cut into the first 3–4 mm of implant-bone contact. The implant explantation was then continued with the torque wrench. After implant removal, the socket was carefully curetted to remove any granulomatous tissue. A new dental implant (Biotechnology Institute S.L, Vitoria, Spain) was placed either immediately or after tissue healing. In the case of delayed implant placement, the socket was grafted with plasma rich in growth factors (PRGF). Preparation of PRGF was performed with KMU 15 kit (Biotechnology Institute S.L, Vitoria, Spain) following the manufacturer instructions. Briefly, blood was collected in 9 ml citrated tubes and centrifuged for 8 min at room temperature [10, 11]. The plasma column just above the Buffy coat was divided into fraction 1 (F1) and fraction 2 (F2). F2 contained the highest platelet concentration (2–3 times higher than peripheral blood), whereas F1 had a similar concentration to peripheral blood. To induce clot formation, 10% calcium chloride solution was added to F1 and F2. The F2 clot was used to fill the socket and F1 was used to prepare a fibrin membrane that covered the surgical area. In the prosthetic phase, definitive transepithelial abutments (BTI Biotechnology Institute) were connected to the implant at the torque recommended by the manufacturer. Impression making and prosthesis connection were performed at the gingival level (transepithelial abutments).

In this study the principal variable of efficacy was the implant survival (the presence of the dental implant in the patient's mouth at the last time follow-up visit). As secondary variables of efficacy, the marginal bone loss and the technical complications were assessed. For the calculation of the marginal bone loss, digital radiographs were used. The linear measurements on the radiograph were calibrated by the known implant length. The mesial and distal distances between the implant platform and the first bone-implant contact were measured at two timepoints. These timepoints were the visit of implant loading (baseline measurement) and the last visit of the patient. The change in the marginal bone level was calculated as the difference between the marginal bone level at baseline and at the last available radiograph. The change in the marginal bone level was expressed as the mean of the change in the mesial and distal bone levels. Implant success was defined according to the criteria suggested by Buser et al. [12]. The health scale of dental implants was also assessed according to the criteria suggested by the International Congress of Oral Implantologists (ICOI) Pisa Consensus Conference [13]. Technical complications such as screw loosening/fracture, ceramic fracture and implant fracture were also assessed.

Demographic data (age and sex), date of implant removal, date of the new implant insertion, implant position, insertion torque, bone type (pristine/regenerated), date of implant loading and type of prosthesis were assessed.

### Statistical analysis

Anonymized database that included the study variables were generated. The qualitative variables were described by calculating the relative/absolute frequency. The continuous variables were described by calculating the mean, standard deviation and the range. The normal distribution of the variables was assessed by the Shapiro–Wilk test. Mann–Whitney test was used to compare the mean marginal bone loss between the implants that were placed in the same surgical session of implant removal and the implants that were placed in a second surgery. The cumulative implant survival rate was calculated with the Kaplan–Meier test. The comparison of the survival rate between immediate or delayed insertion of the replacing implants was performed with the Mantel–Cox test. The statistical significance was set at *p* < 0.05. SPSS v15.0 for Windows statistical software package was used for statistical analysis.

## Results

**Fig. 1:**
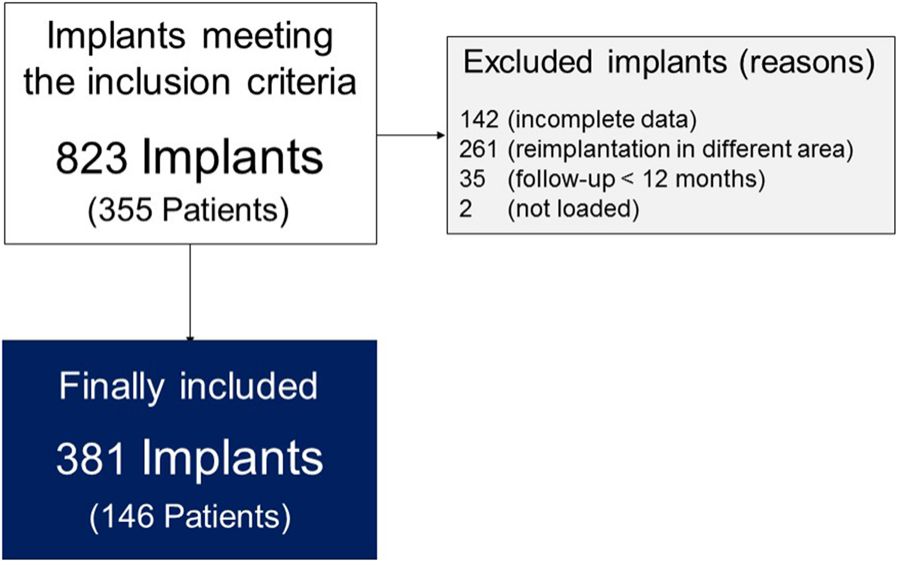
Study flow chart

The anonymized database contained 355 patients and 823 implants. However, 442 implants were excluded as 142 had incomplete data related to study variables, 261 had not been placed in the same region, 2 were inserted before implant removal, 35 had follow-up less than 12 months, and 2 were not loaded (Fig. 1). One hundred and forty six patients had 381 dental implants that were placed in the same position or one-tooth position mesially/distally to the site of explantation. The patients' mean age was 63 ± 10 years (range: 29–84 years). Ninety-seven patients were females and 49 were males. Fifty one (13%) of the implants removed due to the peri-implantitis was placed by the same surgeon who placed the new implants. The other 330 (87%) of the removed implants were placed by other surgeons.

**Fig. 2:**
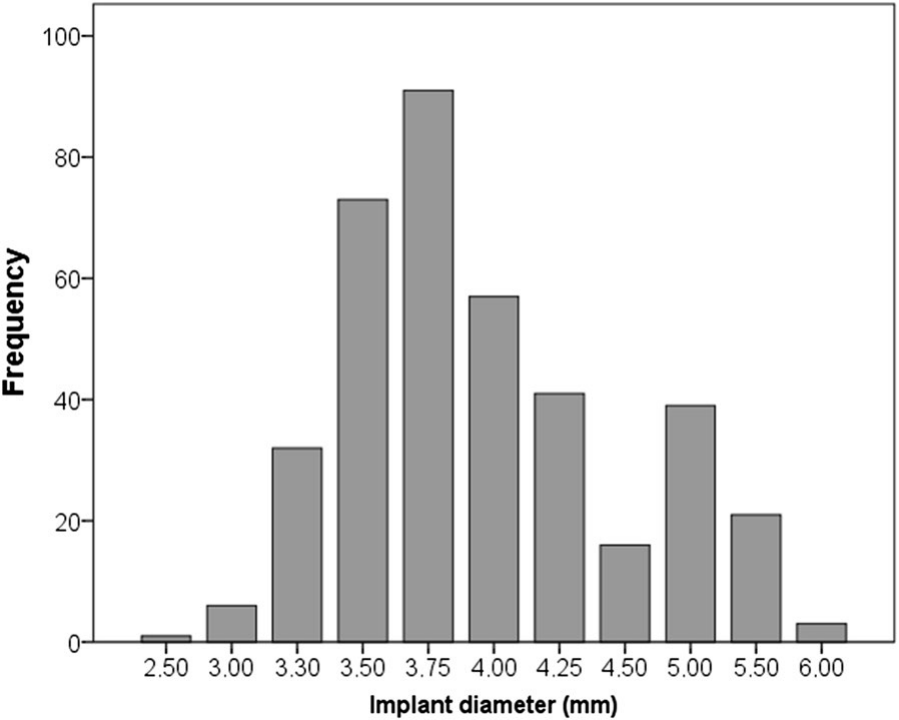
Dental implant diameter

**Fig. 3:**
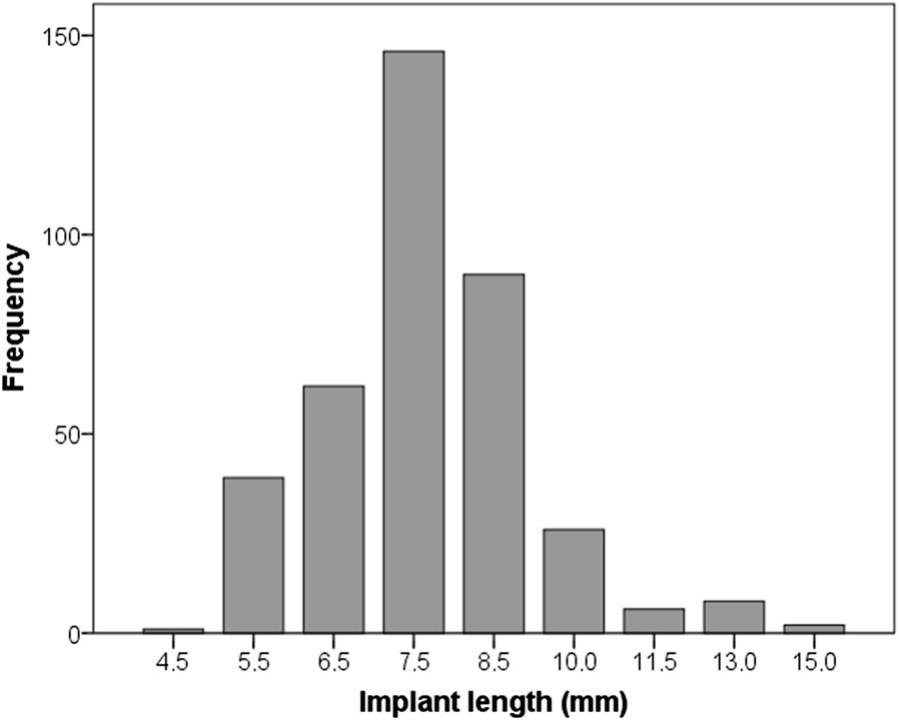
Dental implant length

One hundred sixty four (43%) implants were inserted in the same surgical session as another implant was removed due to peri-implantitis. The other 217 (57%) implants were placed in a second surgical session after implant removal. This second intervention was performed 5 ± 9 months (range: 1–75 months) after implant removal. Overall, 245 (60%) implants were placed in pristine bone (one position mesial/distal to the removed implant) and 136 (36%) in regenerated bone (the same position as the removed implant). Figures 2 and 3 show the distribution of the implants diameter and length, respectively. Two hundred and forty eight (65%) implants were short (length < 8 mm) and 102 were extra-short in length (≤ 6.5 mm). Regarding implant diameter, 112 (29%) implants were narrow (diameter < 3.75 mm). The Mean insertion torque was 44 ± 16 Ncm (range: 5–75 Ncm).

**Fig. 4:**
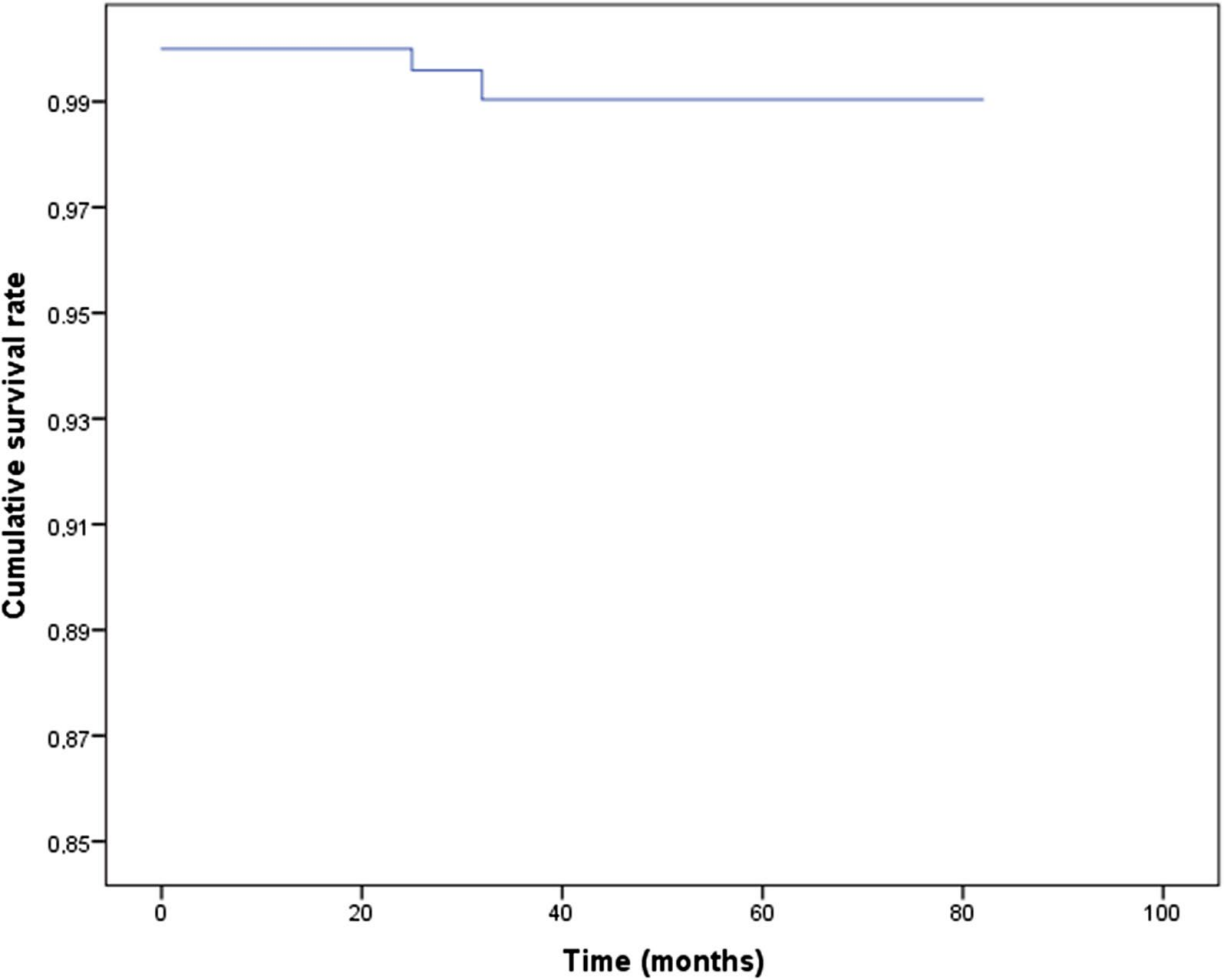
Cumulative implant survival rate

**Fig. 5:**
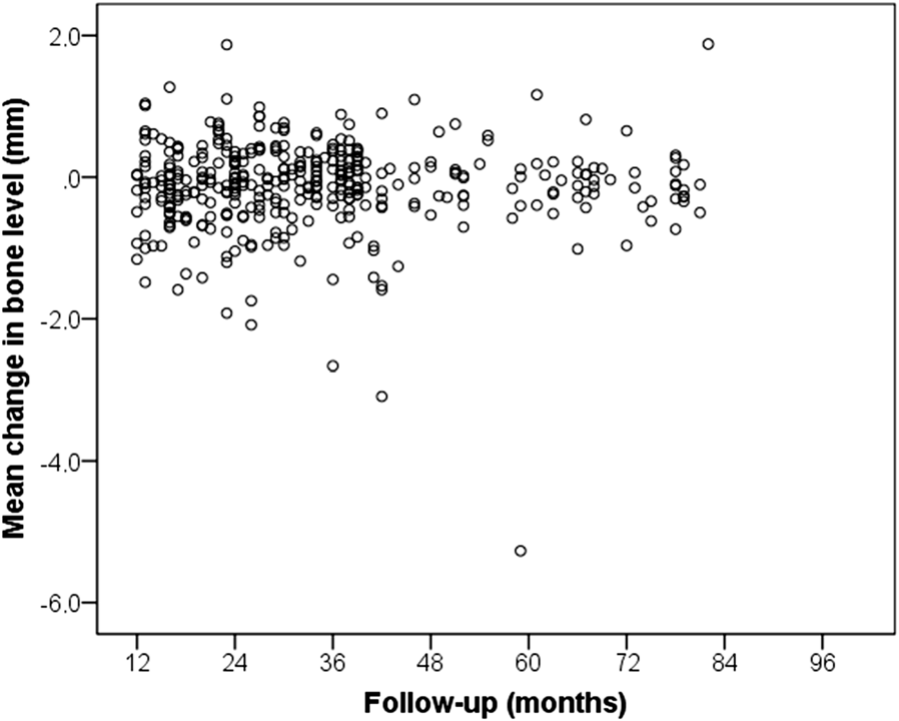
Changes in the level of the marginal follow-up over time. (−) indicates loss and (+) indicates gain

After a mean follow-up of 34 ± 17 months (range: 12–82 months), two implants failed. The cumulative survival rate was 99% (Fig. 4). The marginal bone loss was −0.1 ± 0.6 (range: −5.3 to 1.9 mm). Two hundred and eighteen (57%) implants showed marginal bone loss and 148 implants had it ≤ −0.5 mm (Fig. 5). Only 4 (1%) implants (2 supporting partial prosthesis and 2 complete prosthesis) in 4 patients presented marginal bone loss greater than 2 mm from the basal level. The follow-up time of these implants were 17, 38, 39 and 78 months. For marginal bone loss ≥ 3 mm, 3 implants in 3 patients were affected with follow-up time of 17, 38, and 78 months. One hundred and sixty one (42%) implants had no marginal bone loss. Taking together implant survival and marginal bone loss data, 309 (81%) implants survived and had a marginal bone loss ≤ −0.5 mm. Considering the success criteria suggested by Buser et al. [12] the implant success rate was 99.5%. According to the health scale of dental implants [13], 98.4% of the dental implants had optimal health and had been considered as a success. Replacing the removed implant immediately (same surgical session) or delayed had no significant influence on marginal bone loss (*p* value = 0.839). For the immediate replacement, the marginal bone loss was −0.1 ± 0.7 mm (range: −5.3–1.9 mm). For the delayed replacement, it was −0.1 ± 0.6 (range: −3.1–1.3 mm). Furthermore, no significant differences (*p* value = 0.765) were observed in implant survival (99% for both).

Prosthodontically, 256 (67%) implants were immediately loaded within the first 24 h after insertion. All the implants were screw-retained and restored using transepithelial (transmucosal) abutments. Two hundred and seven implants (54%) supported fixed partial prostheses and 166 (44%) supported fixed complete prosthesis. Seven (2%) implants supported a single crown. The following technical complications were observed: ceramic chipping (3 events), resin tooth fracture (1 event) and prosthetic screw loosening (1 event).

## Discussion

In a recent systematic review by Rakic et al. the prevalence of peri-implantitis has been estimated to be 18.5% at the patient level and 12.8% at the implant level [14]. There is a clinical need for the establishment of effective protocols for the treatment of peri-implantitis that can achieve long-term outcomes. In the study by Carcuac et al. different levels of outcomes have been described [15]. The most strict definition has been further marginal bone loss ≤ 0.5 mm, probing depth ≤ 5 mm and absence of bleeding or suppuration on probing. This has been considered as the peri-implantitis treatment success [16]. Following this definition, treatment success has been only achieved in about one third of the dental implants that were treated for peri-implantitis [15, 17]. Which indicate a clinical need to keep on researching to improve these figures.

However, in the clinical practice, success could be the no progression of the disease and the survival of the dental implant [16]. The success (implant survival + further MBL ≤ 0.5 mm) has been achieved in 57% of the implants after 3 years of follow-up [15]. At 5 years of follow-up, 56.2% of the implants has survived, not retreated or has marginal bone loss ≤ 1 mm [18]. Several factors may influence the success of peri-implantitis treatment. With 5-year follow-up data, it has been observed that the presence of residual deep probing depth, reduced marginal bone level and modified implant surface are factors that have increased the risk of recurrence/progression of the disease [18].

Mardinger et al. have indicated the removal of a failing implant as soon as it is diagnosed as hopeless will improve the chances for reimplantation [19]. This will also prevent further tissue destruction. However, very few reports are available on the clinical outcomes on placing a dental implants in a region previously affected by peri-implantitis [20]. Similar to periodontitis, peri-implantitis is an inflammatory process that is triggered by bacterial plaque and affects peri-implant soft and hard tissue [21]. In patients with periodontitis, implant survival rate has been about 90% [22–24]. Lee et al. have concluded that there is a strong evidence to suggest that periodontitis is a risk factor for implant loss [25].

This study has been conducted to assess the survival and marginal bone loss of dental implants replacing dental implants failed due to peri-implantitis in the context of routine dental practice. The study outcomes have indicated high implant survival rate and marginal bone stability. The cumulative implant survival rate was 99% and the marginal bone loss was −0.1 ± 0.6 (range: −5.3 to 1.9 mm). Only 3 (< 1%) implants presented marginal bone loss ≥ 3 mm after a mean follow-up time of 44 ± 31 months. According to the health scale of dental implants, 98.4% of the dental implants had optimal health [13]. Considering the success criteria suggested by Buser et al. the implant success rate was 99.5% [12]. In another study, immediate implant placement in the same socket of extracted implant due to peri-implantitis has shown high survival rate and marginal bone stability [20]. It has been hypothesized that the decrease in the bacterial load through the removal of the infected implant, the removal of the granulation tissue by adequate socket curettage and the mechanical decontamination of the socket by drilling would enhance the probability of implant survival [20]. The survival of implants immediately placed in infected and non-infected sockets has not shown statistically significant differences [26, 27]. A systematic review has been concluded that implants may be successfully osseointegrated when placed immediately after extraction of teeth due to endodontic and periodontal lesions [28].

In the present study 309 (81%) implants have survived and have shown a marginal bone loss ≤ 0.5 mm. Carcuac et al. have reported the achievement of this outcome in 57% after 3 years of follow-up of surgically treated implants for peri-implantitis [15]. For that, the replacement of dental implant affected by peri-implantitis has resulted in good clinical outcomes in term of implant survival and marginal bone stability.

This study suffers from the limitation of retrospective design, where there is dependency on the availability and accuracy of the clinical records. The absence of a control group limits the extrapolation of the results of this study. However, the study presents the outcome in the context of routine clinical practice, where 381 implants in 146 patients have been assessed. The outcomes of this study justify the performance of a prospective and controlled clinical study to assess the clinical performance of dental implants replacing failed implant due to peri-implantitis.

## Conclusions

When a dental implant affected by peri-implantitis fails, there is a need to replace that implant. Insight of the results of this study, the survival and marginal bone loss of replacement implants support this option for the management of failed implants due to peri-implantitis. Nevertheless new prospective research is needed to confirm these results.

## Data Availability

The data sets used and/or analysed during the current study are available from the corresponding author on reasonable request.
